# A cross-sectional study of the clinical characteristics of hospitalized children with community-acquired pneumonia in eight eastern cities in China

**DOI:** 10.1186/1472-6882-13-367

**Published:** 2013-12-23

**Authors:** Xue-Feng Wang, Jian-Ping Liu, Kun-Ling Shen, Rong Ma, Zhen-Ze Cui, Li Deng, Yun-Xiao Shang, De-Yu Zhao, Li-Bo Wang, Li-Ya Wan, Yi-Qiu Sun, Yan-Ning Li, Zhi-Yan Jiang, Hua Xu, Xin-Min Li, Zhen-Qi Wu, Zhao-Lan Liu, Ying-Hui Hu, Yan Huang, Chun-Hui He, Han Zhang, Yong-Hong Jiang, Hua Liu, Zi Wang

**Affiliations:** 1 Department of Pediatrics, Affiliated Hospital of Liaoning University of Traditional Chinese Medicine, 33 Beiling Street, Huanggu District, Shenyang, Liaoning 110032, China; 2 Centre for Evidence-Based Chinese Medicine, Beijing University of Chinese Medicine, 11 Beisanhuan Dong Road, Chaoyang District, Beijing 100029, China; 3 Department of Respiratory Medicine, Beijing Children’s Hospital, 56 Nanlishi Road, Xicheng District, Beijing 100045, China; 4 First Teaching Hospital of Tianjin University of TCM, 314 Anshan Xi Road, Nankai District, Tianjin 300193, China; 5 Department of Respiratory Medicine, Dalian Children’s Hospital, 154 Zhongshan Road, Xigang District, Dalian, Liaoning 116012, China; 6 Department of Respiratory Medicine, Guangzhou Women and Children’s Medical Center, 318 Renminzhong Road, Yuexiu District, Guangzhou, Guangdong 510623, China; 7 Department of Pediatric Respiratory Medicine, Shengjing Hospital of China Medical University, 36 Sanhao Street, Heping District, Shenyang, Liaoning 110004, China; 8 Department of Pediatric Respiratory Medicine, Nanjing Children’ Hospital, 72 Guangzhou Road, Nanjing, Jiangsu 210008, China; 9 Department of Pediatric Respiratory Medicine, Children’s Hospital of Fudan University, 399 Wanyuan Road, Minhang District, Shanghai 201102, China; 10 Department of Pediatric Respiratory Medicine, Tianjin Children’s Hospital, 225 Machang Road, Hexi District, Tianjin 300074, China; 11 Department of Pediatrics, Jiangsu Provincial Hospital of TCM, 155 Hanzhong Road, Nanjing, Jiangsu 210029, China; 12 Department of Pediatrics, Affiliated Hospital of Shandong University of TCM, 42 Wenhua Xi Road, Jinan, Shandong 250011, China; 13 Department of Pediatrics, Longhua Hospital of Shanghai University of TCM, 725 South Wanping Road, Shanghai 200032, China; 14 Department of Pediatrics, Affiliated Hospital of Guangzhou University of TCM, 16 Baiyun Jichang Road, Guangzhou, Guangdong 510405, China

**Keywords:** Childhood pneumonia, Community-acquired, Clinical characteristics, Treatment, Cross-sectional study, Chinese population

## Abstract

**Background:**

Community-acquired pneumonia in children is common in China. To understand current clinical characteristics and practice, we conducted a cross-sectional study to analyze quality of care on childhood pneumonia in eight eastern cities in China.

**Methods:**

Consecutive hospital records between January 1, 2010 and December 31, 2010 were collected from 13 traditional Chinese medicine (TCM) and western medicine (WM) hospitals in February, May, August, and November (25 cases per season, 100 cases over the year), respectively. A predesigned case report form was used to extract data from the hospital medical records.

**Results:**

A total of 1298 cases were collected and analyzed. Symptoms and signs upon admission at TCM and WM hospitals were cough (99.3% vs. 98.6%), rales (84.8% vs. 75.0%), phlegm (83.3% vs. 49.1%), and fever (74.9% vs. 84.0%) in frequency. Patients admitted to WM hospitals had symptoms and signs for a longer period prior to admission than patients admitted to TCM hospitals. Testing to identify etiologic agents was performed in 1140 cases (88.4%). Intravenous antibiotics were administered in 99.3% (595/598) of cases in TCM hospitals and in 98.6% (699/700) of cases in WM hospitals. Besides, Chinese herbal extract injection was used more frequently in TCM hospitals (491 cases, 82.1%) than in WM hospitals (212 cases, 30.3%) (p < 0.01). At discharge, 818 cases (63.0%) were clinically cured, with a significant difference between the cure rates in TCM (87.6%) and WM hospitals (42.0%) (OR = 9.8, 95% confidence interval (CI): 7.3 ~ 12.9, p < 0.01). Pathogen and previous medical history were more likely associated with the disappearance of rales (OR = 7.2, 95% CI: 4.8 ~ 10.9). Adverse effects were not reported from the medical records.

**Conclusions:**

Intravenous use of antibiotics is highly prevalent in children with community-acquired pneumonia regardless of aetiology. There was difference between TCM and WM hospitals with regard to symptom profile and the use of antibiotics. Intravenous use of herbal injection was higher in TCM hospitals than in WM hospitals. Most of the cases were diagnosed based on clinical signs and symptoms without sufficient confirmation of aetiology. Audit of current practice is urgently needed to improve care.

## Background

Pneumonia is an inflammatory condition of the lung, caused by different pathogens or other etiologic factors [1]. Worldwide it is the leading cause of death in children under five years old [2-4], with nearly 2 million deaths, or almost 19.0% of total deaths in children [5]. In China, it is estimated that there are an annual 21.1 million new cases of pneumonia in children younger than 5 years of age. This is a disease burden second only to India, which has 43 million annual new cases in the same age group [6,7]. In China, pneumonia accounts for most pediatric hospitalizations [8].

Addressing childhood pneumonia remains a difficult public health task in China. In terms of surveillance, pneumonia is not even listed as a national notifiable infectious disease [9], though in 2003 after the outbreak of severe acute respiratory syndrome (SARS), “pneumonia of unknown cause” was required to be reported because airborne etiologic agents such as the SARS coronavirus and human avian influenza virus (H5N1) are extremely pathogenic and capable of quickly causing an epidemic [10]. Moreover, China’s current health policy of “integration of TCM and Western medicine” need large-scale surveys comparing cases of childhood pneumonia at TCM and WM hospitals. Whether for guiding healthcare policy or for improving disease management and prevention, evaluating quality of care in paediatric pneumonia is of paramount importance. Through the ages, WM hospitals hold a principle status in public health. TCM hospitals are gradually into mass vision, although at most time as a complementary role. The two types of hospitals have advantages and disadvantages. Commonly, WM hospitals prefer to acute severe disease, and TCM hospitals prefer to chronic mild disease. To exploring specific difference on pneumonia therapy between two types of hospitals, this cross-sectional study was designed to investigate the distribution, diagnosis, treatment, and clinical outcome of childhood community-acquired pneumonia in China.

## Methods

### Study subjects

Data were drawn from 13 traditional Chinese medicine (TCM) and western medicine (WM) hospitals in eight eastern cities of China: Beijing, Dalian, Guangzhou, Jinan, Nanjing, Shanghai, Shenyang, and Tianjin. Hospitals selection refers to region representation as provincial capitals or coast cities, which reflects a better status of economic and health in China. The hospitals are either affiliated with universities or are provincial/municipal children’s hospitals. Our protocol of study was approved by institutional review board (IRB) of the affiliated hospital of Liaoning University of Traditional Chinese Medicine (principle investigator) which covered each hospital’s IRB.

Children between 1 and 14 years of age presenting to the pediatric ward with clinical diagnosis of community-acquired pneumonia were eligible for the study. Our previous study [11-14] found that admission rates and numbers of severe pneumonia cases, especially in children <1 year of age, between TCM and WM hospitals were highly incongruent. Therefore, to avoid bias in the current study, we excluded children younger than one year old and children with severe pneumonia. Indications for severe pneumonia in patient age 0–3 years were temperature >38.5°C, respiratory rate (RR) >70 breaths/min, chest retractions, nasal flaring, cyanosis, intermittent apnea, grunting, not feeding. Indications for severe pneumonia in patient age 4–14 years were temperature >38.5°C, RR >50 breaths/min, nasal flaring, cyanosis, grunting, and signs of dehydration.

Criteria of diagnosis were based on nationally-recognized guidelines from either the Chinese Medical Association [10] or *Zhu Futang Textbook of Pediatrics*[1], with symptoms of fever, cough and dyspnea, fixed fine moist rales, and patchy infiltrates on chest radiography. Criteria for clinical therapeutic effectivness were also based on national guidelines [15,16]. Clinical cure was defined as temperature ≤37.2°C, disappearance of all symptoms except occasional cough, and disappearance of rales. Clinical improvement was defined as ≤37.2°C, all symptoms and rales reduced. Failure was defined as no improvement in presenting symptoms.

Data sampling was from January 1, 2010 to December 31, 2010. Total study sample was 1300. Consecutive hospitalised records of children with paediatric pneumonia were collected from each hospital in February, May, August, and November, respectively, that is, first consecutive 25 cases each month, or 100 cases per hospital over the 1-year study period.

### Data collection

A predesigned case report form was applied by trained investigators with clinical medicine backgrounds. Information extracted were demographic data including name, gender, age, hospital case number, birth history (route of delivery, birth weight), past medical history (recurrent respiratory tract diseases, eczema, asthma, allergic rhinitis, and other allergies), duration of hospital stay, symptoms and signs up to admission, diagnosis on admission and at discharge, therapy modalities, and clinical outcome at discharge. All records were input into an electronic database based on EpiData software version 3.1.

### Statistical analysis

EpiData software version 3.1 and IBM SPSS software version 17.0 were used for record and data analyses. All *p* values were applied with two-tailed tests and were not adjusted for multiple testing. Logistic regression model was used to explore the relationship between different types of hospitals and the improvement of symptoms.

## Results

### Clinical characteristics

A total of 1298 cases were collected from six TCM hospitals and seven WM hospitals (Table 1). Data of two cases were missing from one hospital. Male to female ratio was 1.41:1. Previous medical history included recurrent pneumonia, recurrent bronchitis, and recurrent upper respiratory tract infection (RURTI) in 349/1298 (26.9%) children; eczema in 130 (10.0%) children; asthma in 53 (4.1%) children; and allergic rhinitis in 56 (4.3%) children. There was significant difference on previous medical history in sampled patients between two types of hospitals.

**Table 1:** Characteristics of children hospitalised with pneumonia in the sampled hospitals

**Characteristics**	**TCM hospitals**		**WM hospitals**		**χ**^**2**^	***P *** **value**
	**Number (n = 598)**	**%**	**Number (n = 700)**	**%**		
Gender					3.75	0.05
Male	333	55.7	427	61.0		
Female	265	44.3	273	39.0		
Age, y					4.74	0.09
1-3	163	27.3	228	32.6		
4-7	327	54.7	346	49.4		
8-14	108	18.0	126	18.0		
Previous medical history						
Recurrent pneumonia^a^	116	19.4	45	6.4	38.60	<0.01
Recurrent bronchitis^b^	43	7.2	13	1.9	5.59	0.02
RURTI^c^	78	13.0	54	7.7	17.33	<0.01
Eczema	73	12.2	57	8.1	2.42	0.12
Asthma^d^	42	7.0	11	1.6	19.52	<0.01
Allergic rhinitis	48	8.0	8	1.1	30.75	<0.01

*Abbreviations*: *TCM* traditional Chinese medicine, *WM* Western medicine, *RURTI* recurrent upper respiratory tract infection, *URTI* upper respiratory tract infection.

^a^Recurrent pneumonia is defined as at least two pneumonia episodes in a 1-year period or at least three episodes over a lifetime, with normal chest radiography between each episode [17].

^b^Recurrent bronchitis is defined as in patients age 0–2 years, three or more bronchitis episodes in 1 year; age 3–14 years, two or more episodes in 1 year [17].

^c^RURTI is defined as in patients age 0–2 years, seven or more episodes of URTI in 1 year; age 3–5 years, six or more episodes in 1 year; 6–14 years, five or more episodes of URTI in 1 year [10,17].

^d^Includes cough-variant asthma.

### Clinical manifestations on admission

Presentation of symptoms and signs on admission in TCM hospitals were, from most to less frequent: cough (99.3%), rales (84.8%), phlegm (83.3%), and fever (74.9%). These percentages were nearly the same as those in WM hospitals: cough (98.6%), fever (84%), rales (75.0%), and phlegm (49.1%). Except for cough (*p* = 0.19), the frequencies of rales, phlegm, and fever were significantly higher in WM hospitals than in TCM hospitals (*p* < 0.01). Average duration of illness from onset of symptoms and signs to admission was markedly higher in WM hospitals than in TCM hospitals (Table 2).

**Table 2:** Symptoms and signs of children with pneumonia on admission to hospital in the sampled hospitals

	**Frequency**		**χ**^**2**^	***P***	**Time from onset of symptoms up to admission (days)**		**Z**	***P***
**Symptoms**	**TCM hospitals (n = 598)**	**WM hospitals (n = 700)**			**TCM hospitals**	**WM hospitals**		
Cough	594	690	1.74	0.19	7.4 ± 6.6	8.8 ± 7.5	−6.11	<0.01
Rales	507	525	18.94	<0.01	1.2 ± 0.7	1.6 ± 1.7	−4.59	<0.01
Phlegm^a^	498	344	164.89	<0.01	5.8 ± 9.7	4.0 ± 6.7	−5.87	<0.01
Fever^b^	448	588	16.52	<0.01	93.6 ± 64.2	147.1 ± 120.7	−8.91	<0.01
Tachypnea	196	87	78.31	<0.01	2.6 ± 2.9	4.9 ± 5.0	−4.72	<0.01
Dyspnea	24	70	17.21	<0.01	2.2 ± 2.3	7.0 ± 15.0	−3.47	<0.01

*Abbreviations*: *TCM* traditional Chinese medicine, *WM* Western medicine.

^a^Includes white sputum, yellow sputum, or difficult to expectorate sputum.

^b^Time of onset of fever up to admission recorded in hours.

### Aetiology diagnosis

Specific pathogens (one pathogen or more) were identified in 624 (48.1%) cases (Table 3). Negative pathogen findings were identified in 524 (40.4%) cases. Eighty-five (6.5%) cases were not tested for pathogens. Data were missing in 65 (5.0%) cases. TCM hospitals performed significantly fewer pathogen tests as compared to WM hospitals (30.3% versus 69.7%, p < 0.01). In terms of pathogenic agent, *Mycoplasma pneumoniae* (*M pneumoniae*) was identified more frequently, followed by mixed pathogens.

**Table 3:** Etiologic agents identified in children hospitalised with pneumonia in the sampled hospitals

**Pathogen**	**1-3 years (N = 391)**				**4-7 years (N = 673)**				**8-14 years (N = 234)**				**Total**
	**TCM hospitals**		**WM hospitals**		**TCM hospitals**		**WM hospitals**		**TCM hospitals**		**WM hospitals**		
	**n**	**%**	**n**	**%**	**n**	**%**	**n**	**%**	**n**	**%**	**n**	**%**	
Bacteria^a^	1	0.7	32	15.6	4	1.2	37	11.6	1	0.9	7	6.2	82
*M pneumoniae*^b^	37	22.8	36	17.6	89	27.2	59	18.6	39	36.1	39	34.5	299
Virus^c^	0	0.0	25	12.2	4	1.2	23	7.2	0	0.0	4	3.6	56
Mixed^d^	0	0.0	41	20.0	5	1.5	101	31.8	1	0.9	39	34.5	187
Negative finding^e^	95	58.6	68	33.2	183	56.0	96	30.2	58	53.7	24	21.2	524
Not tested^f^	29	17.9	3	1.4	42	12.8	2	0.6	9	8.4	0	0.0	85
Total	162		205		327		318		108		113		1233^g^

*Abbreviations*: *TCM* traditional Chinese medicine, *WM* Western medicine.

^a^Identified through sputum or blood culture.

^b^Identified through serum antibody or antigen test.

^c^Identified through nasopharyngeal secretion antibody test or serum antibody test.

^d^Infection of two or more pathogens.

^e^None identified through above tests.

^f^Not go through any etiological tests.

^g^Missing 65 cases of etiological tests results.

### Treatment

Prevalence of intravenous antibiotic use in TCM and WM hospitals were 595 (99.3%) and 699 (98.6%), respectively, in records of children with pneumonia, with no significant difference (*p* = 0.34) (Table 4). However, the patterns of antibiotic use in TCM and WM hospitals were different. In TCM hospitals, antibiotics used were by frequency, macrolides, third generation cephalosporins, second generation cephalosporins, first generation cephalosporins, and β-lactamases. In WM hospitals, antibiotics used were by frequency, third generation cephalosporins, macrolides, second generation cephalosporins, β-lactamases, and first generation cephalosporins. Oral antibiotic use was significantly higher in WM hospitals (243 cases) than in TCM hospitals (65 cases) (*p* < 0.01). Chinese herbal injections were applied more frequently in TCM hospitals (491 cases, 82.1%) than in WM hospitals (212 cases, 30.3%) (*p* < 0.01). Use of Chinese proprietary medicines was similar across both types of hospitals (40.0% in TCM hospitals versus 42.2% in WM hospitals) (*p* = 0.37). The most frequently used herbal injections in TCM hospitals were Tanreqing, Danshen, Shengmai, and Xiyanping. While in WM hospitals they were Xiyanping and Xixinnao. In addition, 312 (52.2%) children were treated with herbal decoctions consisting of several herbs and 320 (53.5%) underwent external application of herbs.

**Table 4:** Treatment modalities in children hospitalised with pneumonia in the sampled hospitals

**Medications**	**TCM hospitals**		**WM hospitals**		**χ**^**2**^	***P *** **value**
	**Number (n = 598)**	**%**	**Number (n = 700)**	**%**		
Antibiotics						
IV	595	99.5	699	99.9	-	0.34
Macrolide	383	64.0	357	51.0	22.40	<0.01
Third generation cephalosporin	186	31.1	390	55.7	79.13	<0.01
Second generation cephalosporin	114	19.1	186	26.6	10.23	0.01
First generation cephalosporin	98	16.4	89	12.7	3.53	0.06
β-lactamase inhibitor	73	12.2	101	14.4	1.37	0.24
Oral	65	10.9	243	34.7	101.32	<0.01
Inhalation^a^	8^a^	1.3	0	0.0	-	<0.01
Antiviral drugs						
IV	10	1.7	77	11.0	44.87	<0.01
Inhalation	41	6.9	43	6.1	0.27	0.60
β2 agonist						
Oral	205	34.3	216	30.9	1.73	0.19
Inhalation	342	57.2	205	29.3	102.99	<0.01
Steroids						
IV	61	10.2	39	5.6	9.72	0.02
Inhalation	188	31.4	129	18.4	29.57	<0.01
TCM treatment						
Chinese herbal extract injection	491	82.1	212	30.3	348.83	<0.01
Chinese patent drugs	239	40.0	297	42.2	0.81	0.37
Decoction	312	52.2	16	2.3	425.03	<0.01
External methods	320	53.5	179	25.6	106.39	<0.01

*Abbreviations*: *TCM* traditional Chinese medicine, *WM* Western medicine.

^a^7 cases of gentamicin and 1case of erythrocin.

### Clinical outcome at discharge

Eight hundred and eighteen cases were identified as clinically cured when discharged, a cure rate of 63.0% (Tables 5 and 6). Relief rates of fever, though, were significantly higher in TCM hospitals than in WM hospitals (OR = 3.5, 95% CI: 2.0 ~ 6.3, *p* < 0.01), but the time from onset of fever to complete resolution was similar in two types of hospitals (*p* = 0.013). Relief rates of cough and rales were significantly higher in TCM hospitals than in WM hospitals (OR = 2.6, 95% CI 2.6 ~ 3.2, OR = 4.1, 95% CI: 3.0 ~ 5.7, *p* < 0.01), but duration from onset to complete relief of cough and rales was longer in TCM than in WM hospitals (*p* < 0.01). Relief rates of phlegm were significantly higher in WM hospitals than in TCM hospitals (OR = 0.6, 95% CI 0.4 ~ 0.8, *p* < 0.01), and the time from onset of phlegm to complete resolution was longer in TCM than in WM hospitals (*p* < 0.01). There was no difference in relief rates of tachypnea (OR = 1.2, 95%CI: 0.5 ~ 2.8, *p* = 0.66) between two types of hospitals. The data of dyspnea did not match criteria of logistic regression. The results were adjusted for gender, age, pathogen and previous medical history listed in Table 1 and Table 3. Relief rates of rales were significantly higher (OR = 7.2, 95% CI 4.8 ~ 10.9) after adjustification. Duration of hospitalization was significantly longer in WM hospitals than in TCM hospitals (*p* < 0.01). There was marked difference in treatment efficacy between TCM hospitals and WM hospitals (OR = 9.8, 95% CI 7.3 ~ 12.9, *p* < 0.01).

**Table 5:** Symptom outcomes of children hospitalised with pneumonia in the sampled hospitals

	**TCM hospitals**	**WM hospitals**	**OR**^**b**^	**95% CI**^**c**^	**P**^**d**^	**AOR**^**e**^	**95% CI**^**f**^	**AP**^**g**^
**Fever**								
Cured	433	524						
Failed	15	64	3.5	2.0 ~ 6.3	<0.01	3.7	2.0 ~ 6.8	<0.01
**Cough**								
Cured	325	220						
Failed	269	470	2.6	2.6 ~ 3.2	<0.01	2.5	2.0 ~ 3.1	<0.01
**Phlegm**								
Cured	291	247						
Failed	209	104	0.6	0.4 ~ 0.8	<0.01	0.4	0.2 ~ 0.6	<0.01
**Tachypnea**								
Cured	179	78						
Failed	17	9	1.2	0.5 ~ 2.8	0.7	1.7	0.5 ~ 6.0	0.4
**Rales**								
Cured	446	335						
Failed	61	190	4.1	3.0 ~ 5.7	<0.01	7.2	4.8 ~ 10.9	<0.01
**Symptoms**								
Cured	524	294						
Improved	72	403						
Failed^a^	2	3	9.8	7.3 ~ 12.9	<0.01	9.7	7.3 ~ 13.1	<0.01

^a^5 cases experienced no improvement of symptoms and were voluntarily discharged.

^b^odds ratio.

^c^odds ratio confidence interval.

^d^P value.

^e^The odds ratio of adjusted gender, age, pathogen, previous medical history.

^f^The odds ratio confidence interval of adjusted gender, age, pathogen, previous medical history.

^g^The P value of adjusted gender, age, pathogen, previous medical history.

**Table 6:** Overall clinical outcome of children hospitalised with pneumonia in the sampled hospitals

**Symptoms**	**Outcome at discharge**^**a**^		**χ**^**2**x^	***P***	**Time from admission to recovery from symptoms (days)**		**Z**	***P***
	**TCM hospitals (n/N)**	**WM hospitals (n/N)**			**TCM hospitals**	**WM hospitals**		
Fever^b^	433/448	565/588	0.23	0.63	53.2 ± 51.5	63.5 ± 63.0	−2.49	0.01
Cough	325/594	220/690	68.10	<0.01	9.5 ± 4.9	8.2 ± 3.5	−3.01	<0.01
Phlegm^c^	292/498	244/344	13.30	<0.01	6.5 ± 9.1	3.1 ± 5.4	−7.55	<0.01
Tachypnea	179/196	78/87	0.20	0.65	2.4 ± 2.0	3.4 ± 2.3	−4.67	<0.01
Dyspnea	24/24	69/70	-	1.00	3.3 ± 2.2	3.8 ± 2.5	−0.68	0.50
Rales	37/507	174/525	105.92	<0.01	1.4 ± 1.6	1.7 ± 1.9	−9.07	<0.01
Duration of hospital stay	-	-	-	-	8.7 ± 5.8	9.0 ± 3.8	−3.44	<0.01

*Abbreviations*: *TCM* traditional Chinese medicine, *WM* Western medicine.

^a^Symptom almost disappeared.

^b^Time of onset of fever up to admission recorded in hours.

^c^Includes white sputum, yellow sputum, or difficult to expectorate sputum.

## Discussion

This cross-sectional study is the first of its kind investigating the clinical profile of children hospitalized with community-acquired pneumonia in China. Our sample was drawn from children of different ages admitted with pneumonia to 13 hospitals across eight eastern cities in China. The consecutive hospital charts covered four seasons. We believe the information gathered reflects current clinical characteristics of childhood CAP in China.

Children with histories of respiratory disease or allergy accounted for 41.0% of the 1298 cases. The percentage (12.4%) of childhood pneumonia cases with histories of recurrent pneumonia was high, similar to study findings elsewhere [18,19]. Ozdemir and colleagues [20] in their sample of 595 hospitalized children found that 62 (10.4%) patients had histories of recurrent pneumonia. In our study, 10.2% of admitted children had recurrent upper respiratory tract infection (RURTI). Risk factors for RURTI are known to be atopy (allergy), daycare attendance, tobacco smoke or air pollution exposure, among others [21]. Such correlations for our sample need further corroboration.

In our study, pneumonia patients with atopic condition were common. Percentages of children with histories of eczema, asthma, or allergic rhinitis were 10.0%, 4.1%, and 4.3%, respectively. Bronchial pneumonia was a complication of all asthma cases. There were also cases of undiagnosed asthma among patients with recurrent pneumonia. This seems to concur with other authors’ findings. For example, Ozdemir and colleagues [20] reported that 30.6% of pneumonia inpatients had histories of asthma. Youn and Lee [22] found that history of asthma might increase the risk of pneumonia caused by *Mycoplasma pneumoniae*. Our study found that onset of childhood pneumonia is closely associated with medical history, in particular with respiratory or atopic conditions. Therefore, attention should be focused on such children and preventive measures applied such as immunization [23,24] and educating families about avoiding contact with allergens and other triggers.

Significant difference existed between TCM and WM hospitals in the presenting symptoms and signs at admission. The World Health Organization’s (WHO) guideline on the management of childhood pneumonia [25] has been widely adopted in developing countries. The guideline suggests that cough and dyspnea present high sensitivity and specificity as main indicators for pediatric pneumonia diagnosis. However, in China dyspnea is rarely applied to diagnose mild pediatric pneumonia because though respiratory rate is increased, it is still within normal range, especially in very young children. Instead, cough and auscultatory findings are more commonly used. In our study, the prevalence of cough at admission was similar between TCM hospitals and WM hospitals. These data are concordant with WHO data [25]. Although there was significant difference between the two types of hospitals regarding other symptoms and signs, a large proportion of children presented with fever and rales.

Duration of symptoms and signs in children before admission in WM hospitals was markedly longer than in children in TCM hospitals. We suspect that the two types of hospitals differ in regard to criteria for admission (such as severity of disease), ability of the family to afford hospitalization, or bed availability. Further studies are required to explore relevant factors that influence hospital intake.

With the growing use of antibiotics in China, incidence of childhood bacterial pneumonia stabilized and declined between 1985 and 2008 [8]. But pneumonia caused by other pathogens increased, especially respiratory syncytial virus and mixed bacterial and viral infections. As elsewhere in the world, China has seen a rise in pneumonia caused by *M pneumoniae* in school-age children [26,27]. Our study showed that *M pneumoniae* accounted for 23.0% of all cases and was highest in preschool children, who comprised 30.8% of all *M pneumoniae* cases. Our previous randomised controlled trial [11] found that *M pneumoniae* pneumonia showed highest prevalence in preschool children, accounting for 43.6% of all cases. Studies in some countries, such as Korea, also found that *M pneumoniae* pneumonia was prevalent in 4 to 6 year-old children, reaching almost 60% [22,27,28]. Current evidence indicates that *M pneumoniae* pneumonia is appearing in increasingly younger children.

In our study, mixed pathogen infection was found in 14.4% of cases and was highest in preschool children at 22.0%. Some authors report that age is related closely to pathogen type in mixed infections, with *M pneumoniae* as one of the most common pathogens in such infections [29-31]. Our study found mixed infections with *M pneumoniae* accounting for the highest proportion of such infections of which the majority of cases were preschool children.

Pediatric pneumonia can be considered a syndrome [32], thus comprehensive treatment and management are needed [10,23]. In China, treatment of childhood pneumonia involves myriad WM and TCM methods. In both types of hospitals, common modalities include intravenous injection of antibiotics and intravenous injection of Chinese herbal preparations. External therapy is also widely applied, mainly inhalation therapy in WM hospitals and external application of herbal medicine and cupping therapy in TCM hospitals.

Antibiotics were the mainstay of childhood pneumonia treatment in the 13 hospitals included in our study. Other authors [33-35] have reported that macrolides are more effective in shortening disease duration and reducing severity of *M pneumoniae* pneumonia compared with other antibiotics. Macrolides are also more effective for a wide range of bacterial pathogens [33,35], which might be one of the reasons that in our study this class of antibiotics was widely used. However, macrolide resistance is a serious worldwide problem [36-38]. Suzuki and colleagues [35] reported that of 96 cases of *M pneumoniae* pneumonia in Yamagata, Japan, 83.0% were identified as macrolide-resistant. Cephalosporins are widely used for treating childhood pneumonia in China. In fact, there has been inappropriate use and abuse of cephalosporins in China [39,40], where cephalosporins use was higher than Northern England (36% in 542 children [41]). In cases of pneumonia caused by *M pneumoniae,* cephalosporins are ineffective. In our study, among 1298 cases, 576 patients (44.4%) received third generation cephalosporins and 300 patients (23.1%) received second generation cephalosporins. In general, selection of antibiotics in China has not been based on drug sensitivity testing but on clinician experience or medical insurance policy. With the promulgation by the Chinese Ministry of Health in August 2012 of regulations on the rational use of antibiotics [42], it is hoped that arbitrary administration of antibiotics, especially for childhood pneumonia, will be curbed.

In the modernization drive, TCM injection in China was popularly used in both types of hospitals. There was a lot of literatures [43-50] reported that the effect of combined therapy with these injections plus conventional treatment for pediatric pneumonia was better than conventional treatment alone. Therefore, this could be explained as the reasons for their use. However, the adverse reaction must not to be overlooked as herb-drug interaction might exist. According to literature review, most common adverse reactions of Chinese herbal injections were rash and pruritus, diarrhea, or vomiting [51]. The high incidence of adverse reaction may be related to physiological characteristics of children or irrational use and preparing technology. Thus, appropriate measures should be taken to reduce the occurrence of such events.

Length of hospitalization in our study was found to be shorter in TCM hospitals than that in WM hospitals. This may be related to the mildness of disease or turnover in ward beds as required by local health policy. Another possible reason for shorter hospital stay in TCM hospitals is that treatment that combines TCM and WM based on syndrome differentiation is more effective than WM alone in resolving symptoms, thus reducing disease duration. In our study, fewer patients in TCM hospitals than in WM hospitals were reported as having symptoms, such as cough and sputum, at discharge. Shorter hospital stay in TCM hospitals may also be attributed to lower emphasis on strict adherence to discharge guidelines. For example, patients deemed “cured” and who are therefore discharged in TCM hospitals would in WM hospitals be considered only “improved” and have a longer length of stay.

Treatment approach differs between TCM and WM. TCM treatment is based on syndrome differentiation in which individual signs and symptoms are evaluated and treated together holistically instead of the solitary symptom approach of WM. With pneumonia, TCM considers sputum an essential sign as part of the overall syndrome. Therefore, TCM clinicians focus on the presence of sputum by observation of signs or airway suctioning. Treatment is rendered based on the quantity, quality, and color of sputum. WM clinicians, on the other hand, view these finer points of sputum as less important, and assess and treat based simply on whether or not sputum is present. Indeed in our study, description of sputum was lacking in most of the cases in WM hospitals. TCM hospitals showed an advantage in relieving rales. This may be because more external therapy, such as herbal medicine application and cupping, was used in TCM hospitals.

The disappearance of rales may be associated with pathogen and previous medical history. Some pathogen induced pneumonia such as *M pneumoniae* pneumonia need more time to absorb inflammation, so rales usually lasted longer than the others. Children with recurrent respiratory disease were more susceptible to illness. Once they got pneumonia, they need more time to recovery than health children. Thus, we should pay more attention to such children and prevent them from pneumonia.

Another difference between TCM and WM hospitals in terms of treatment approach is aetiology diagnosis. As mentioned, TCM practitioners render treatment based on syndrome differentiation. As such, we suspect pathogen testing plays a less important role in guiding treatment than for WM practitioners. This may be the reason that in our study, pathogen testing was ordered less frequently in TCM hospitals than in WM hospitals. (National guidelines in China recommend pathogen testing for CAP [1,10]).

Criteria for evaluating therapeutic effectiveness in childhood pneumonia between TCM and WM hospitals were about the same. But weighting of individual parameters was different. For example, based on guidelines [1,10], fever normalization is foremost for assessing whether pneumonia is only improved or cured. Improvement of symptoms, such as cough, is used to evaluate the status of disease (cured, clinical improvement, or failed). Clinical cure of disease is based on the disappearance of rales (moist rales) through auscultation.

A limitation of our study is that this was a cross-sectional study. Our data was extracted from medical records at 13 hospitals. Although we exercised quality control to the best of our ability during the extraction process, still the quality and scope of the study were dependent on these records.

Another limitation is that patients in this study were drawn from eight cities in China. The data are not representative of childhood CAP elsewhere in China, especially non-urban areas where healthcare seeking behavior and delivery practices vary widely from those in metropolitan locales.

## Conclusions

Our study presents the clinical characteristics of hospitalized children with pneumonia in eight eastern cities in China. Intravenous use of antibiotics was highly prevalent regardless of causative organism. As expected, intravenous use of herbal injections was more prevalent in TCM hospitals than in WM hospitals. This study finds that the basic characteristics of children with pneumonia admitted to the included TCM and WM hospitals are similar. Universal issues, such as inconsistent application of diagnostic criteria and treatment based on clinical experience instead of current guidelines, exist in both types of hospitals. Awareness of these and other shortcomings and taking necessary measures to overcome will help clinicians in China improve their care practice for children with pneumonia.

## Competing interests

The authors declare that they have no competing interests.

## Authors’ contributions

WXF designed and revised the manuscript and organized the study. LJP designed, revised the manuscript and provided methodology perspectives. Other authors including SKL, MR, CZZ, DL, SYX, ZDY, WLB, WLY, SYQ, LYN, JZY, XH, LXM, WZQ, HYH, HY, HCH, ZH, JYH and LH contributed to implementation of the survey, LZL and WZ were responsible for data analyses. All authors read and approved the final manuscript.

## Pre-publication history

The pre-publication history for this paper can be accessed here:

http://www.biomedcentral.com/1472-6882/13/367/prepub
